# The Impact of Donor RBC Storage Duration on the Efficacy of Chronic RBC Exchange

**DOI:** 10.1002/jca.70163

**Published:** 2026-08-05

**Authors:** Jaehyup Kim

**Affiliations:** ^1^ Department of Pathology, Division of Transfusion Medicine and Hemostasis University of Texas Southwestern Medical Center Dallas Texas USA

**Keywords:** hemoglobinopathy, RBC exchange, sickle cell disease, transfusion

## Abstract

Red blood cells (RBCs) accumulate damage during storage, shortening their post‐transfusion lifespan. For chronic RBC exchange (RBCx), fresh RBCs have been preferred because of this concern. This study assessed the impact of RBC storage time and analyzed 20 procedures using freshness‐waived RBCs. The results confirmed a reduction in calculated RBC lifespan. However, the impact on hematocrit or HbS percentage was not significant. Extending RBC freshness criteria for chronic RBCx could reduce costs while maintaining similar outcomes.

## 1 Introduction

Longer storage duration of red blood cells (RBCs) is associated with oxidative damage and metabolic changes, which contribute to reduced persistence after transfusion [1, 2, 3, 4, 5]. Given concerns about reduced RBC quality and survival, fresh RBC units have been advocated for chronic RBC exchange (RBCx) in patients with sickle cell disease to maximize benefits. However, the impact of RBC storage duration on chronically transfused patients with sickle cell disease remains unclear, and there is no standard policy on the freshness of RBCs for exchange [6]. This retrospective cohort study sought to determine whether using RBC units with longer storage duration affects subsequent RBC persistence after RBCx, the percentage of hemoglobin S, or hematocrit.

## 2 Materials and Methods

This retrospective cohort study was conducted at the University of Texas Southwestern Medical Center Clements University Hospital, and Parkland Memorial Hospital, both tertiary care hospitals, in Dallas, Texas. The study was approved by the University of Texas Southwestern Medical Center Institutional Review Board (STU‐122015‐030). Between January 2024 and January 2026, the number and age of RBC units at the time of exchange were recorded for RBCx patients rescheduled to a later date when the allocated RBC units were expected to be more than 10 days old. Laboratory findings (% HbA, % HbS, hematocrit) before and after RBCx, along with the procedure date, were collected through retrospective chart review. RBC life spans were calculated using the previously described method for procedures performed with freshness‐waived RBC units and for procedures performed with RBC units meeting the freshness policy within a one‐year period [7]. The calculated RBC life expectancy, the interval between RBCx, and changes in HbS percentage and hematocrit between RBCx were compared using a two‐tailed paired *t*‐test.

## 3 Results

**FIGURE 1:**
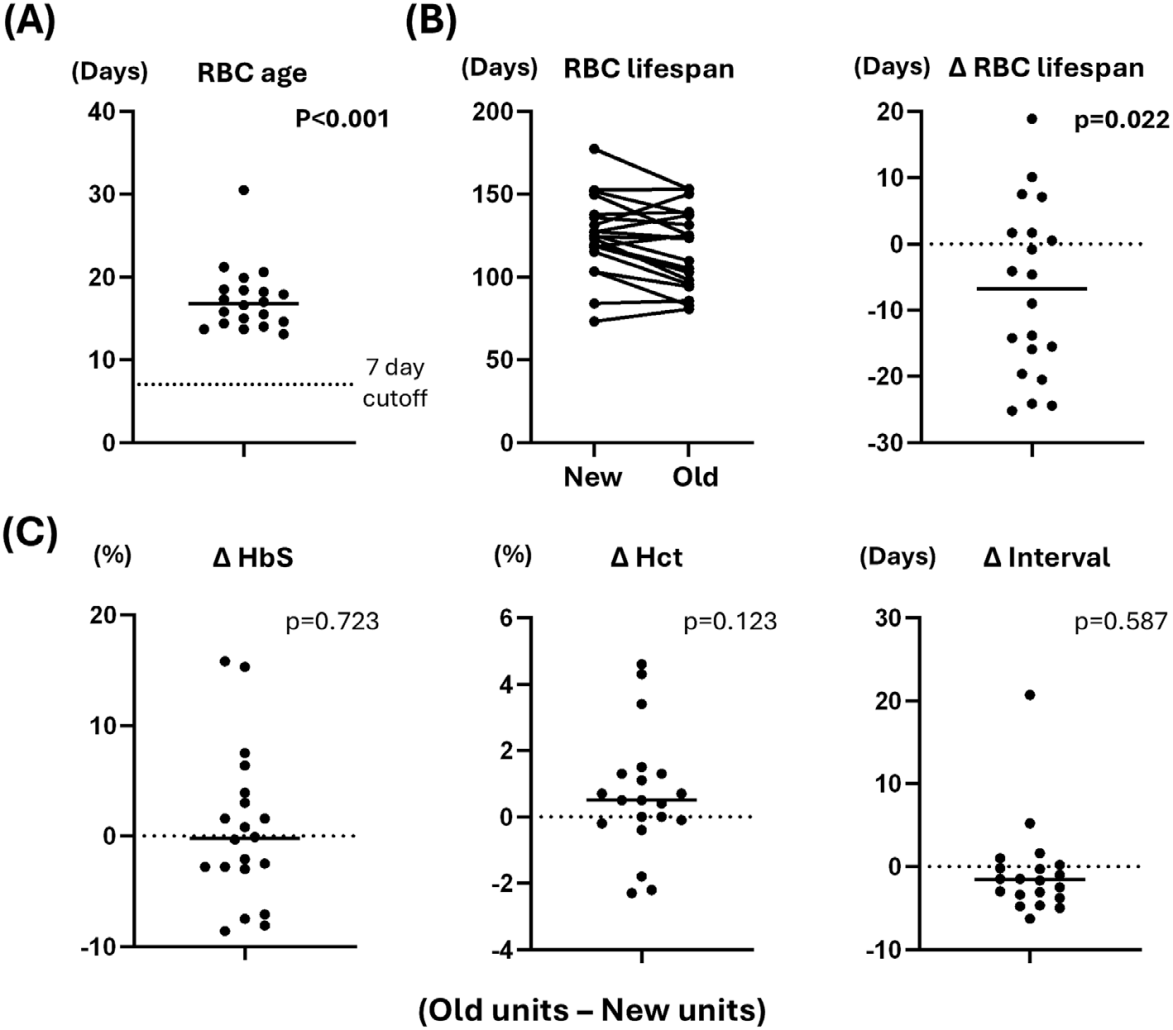
The impact of RBC storage duration on calculated RBC lifespan and clinical parameters. (A) The distribution of RBC storage duration for 20 procedures included in the current study. (B) Comparison of calculated RBC lifespan from RBCx using fresh and old RBC units and the difference in calculated RBC lifespan for each patient. (C) Differences in increase in HbS between RBCx, decrease in hematocrit between RBCx, and days until next RBCx. Average values from RBCx using new RBC units were subtracted from those calculated from RBCx using old RBC units.

During the study period, 43 procedures were performed with freshness‐waived RBCs. Twenty‐three procedures were excluded from the analysis because not all of the units required waiver, lab values were missing, the number of procedures using fresh units was insufficient to establish a baseline, or the same patient required repeat freshness waiver. The average RBC storage duration was 17.3 ± 3.9 days across 20 procedures from 20 patients included in the analysis, which was statistically different from a 7‐day cut‐off (*p* < 0.0001) or a 10‐day automatic waiver limit (*p* < 0.0001) (Figure 1A). For each patient, RBC lifespan was calculated for procedures performed during the study period using the previously reported method [7]. The calculated RBC lifespan was 117.8 ± 23.7 days for freshness‐waived (old) RBC units (Figure 1B). For comparison, the calculated RBC lifespan from procedures performed using non‐waived (new) RBC units over a 1‐year period was collected and averaged, which was 125.0 ± 23.7 days (Figure 1B). There was a statistically significant reduction in calculated RBC lifespan for old RBC units (*p* = 0.022) (Figure 1B). The old RBC group showed a non‐significant increase in HbS% until the next RBCx (0.55 ± 6.83 percent points, *p* = 0.723) or a decrease in hematocrit (0.67 ± 1.84 percent points, *p* = 0.123) (Figure 1C). The interval between RBCx was also not statistically different (−0.70 ± 5.70 days, *p* = 0.587) (Figure 1C).

## 4 Discussion

This study found a negative correlation between RBC storage duration and the lifespan of transfused RBCs in patients with SCD, but no such correlation was found for changes in HbS% or hematocrit. The calculated RBC lifespan of about 120 days, compared with the average RBCx interval of 38 days, means that most transfused donor RBCs will be replaced before they reach the end of their lifespan.

Since patients receiving chronic RBCx require multiple phenotype‐matched units, relaxing the freshness requirement could provide considerable benefits. For the 20 procedures included in the analysis, 194 RBC units received a freshness waiver, with an average of 2.8 antigen‐matching requirements per unit. Four patients had prior alloimmunization, and the remaining patients received Rh and K matching to prevent alloimmunization. The estimated cost saving was 44 160 USD, or 228 USD per RBC unit allocated for the procedures included in the analysis, based on the earlier report of 80 USD per antigen matching [8].

Limitations of this study include a relatively small number of cases from a single medical center, and a relatively low average storage duration of freshness‐waived units at 17.3 ± 3.9 days, close to the common 15‐day cutoff [6]. Despite these limitations, the results suggest that the potentially reduced survival of donor RBCs with longer storage duration may not significantly affect clinical response in patients receiving chronic RBCx. This raises the question of whether strictly adhering to the RBC freshness requirement for patients receiving chronic RBCx provides sufficient benefit to justify the added strain on the RBC supply and the increased cost.

## Author Contributions

Jaehyup Kim was responsible for the conceptualization of the research, research design, data collection, data analysis, and manuscript writing.

## Funding

The author has nothing to report.

## Conflicts of Interest

The author declares no conflicts of interest.

## Data Availability

The data that support the findings of this study are available from the corresponding author upon reasonable request.
